# Speeding up biomolecular interactions by molecular sledding†
†Electronic supplementary information (ESI) available: Synthesis procedures, experimental procedures and Fig. S1–S11. See DOI: 10.1039/c5sc03063c



**DOI:** 10.1039/c5sc03063c

**Published:** 2015-10-07

**Authors:** Alexander Turkin, Lei Zhang, Alessio Marcozzi, Walter F. Mangel, Andreas Herrmann, Antoine M. van Oijen

**Affiliations:** a Single-molecule Biophysics , Zernike Institute for Advanced Materials , University of Groningen , Groningen 9747 AG , The Netherlands . Email: a.m.van.oijen@rug.nl; b Department of Polymer Chemistry , Zernike Institute for Advanced Materials , University of Groningen , Groningen 9747 AG , The Netherlands . Email: a.herrmann@rug.nl; c Brookhaven National Laboratory , Upton , NY 11973-5000 , USA

## Abstract

Numerous biological processes involve association of a protein with its binding partner, an event that is preceded by a diffusion-mediated search bringing the two partners together. Often hindered by crowding in biologically relevant environments, three-dimensional diffusion can be slow and result in long bimolecular association times. Similarly, the initial association step between two binding partners often represents a rate-limiting step in biotechnologically relevant reactions. We demonstrate the practical use of an 11-a.a. DNA-interacting peptide derived from adenovirus to reduce the dimensionality of diffusional search processes and speed up associations between biological macromolecules. We functionalize binding partners with the peptide and demonstrate that the ability of the peptide to one-dimensionally diffuse along DNA results in a 20-fold reduction in reaction time. We also show that modifying PCR primers with the peptide sled enables significant acceleration of standard PCR reactions.

## Abstract 2

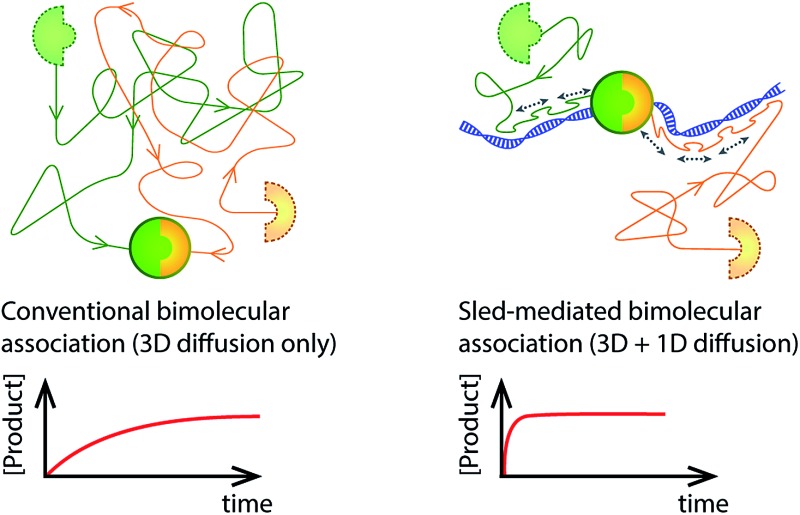
Reaction partners are functionalised with a DNA sliding peptide and the association between them is significantly speeded up in the presence of DNA in solution.

## Introduction

The crowded intracellular environment presents many challenges for basic molecular processes to occur. Non-specific interactions between proteins hinder diffusional mobility and increase the time needed for binding partners to find each other and associate.^1^ Nature displays several examples in which the dimensionality of search processes is reduced to speed up association times.^2^ For example, binding partners of certain classes of cell-surface receptors associate with lipid membranes and utilise two-dimensional diffusion to promote association.^3^ Many DNA-interacting proteins find specific sequences or lesions in large amounts of nonspecific DNA by performing one-dimensional random walks along the DNA.^4^ Every time such a protein associates with DNA, it transiently diffuses along the duplex and thus drastically increases the number of sampled DNA positions per unit of time. It then dissociates from the DNA, undergoes three-dimensional (3D) diffusion through solution to rebind at an entirely different region and again searches a stretch by one-dimensional (1D) diffusion. The combination of 3D and 1D searches gives rise to a drastic increase in the effective bimolecular association rate constant of the protein with its target.^5,6^

An example of a naturally occurring system in which 1D diffusion along DNA is used to speed up association between two proteins is found in adenovirus.^7,8^ During viral maturation, a large number of proteins within a single viral particle need to be proteolytically processed by the adenovirus protease (AVP) before infection of a cell.^9^ Tight packing of protein and DNA within the viral particle makes regular 3D diffusion as a mechanism for the protease to travel from one target to the other impossible. Recent work has shown that the AVP protein^10^ recruits the short 11-a.a. pVIc peptide (GVQSLKRRRCF),^11^ itself a proteolytic product in early maturation, and uses it to slide along the DNA inside the viral particle and thus effectively reduces the search space for the protease from three dimensions to one.^8^

## Results and discussion

**Fig. 1:**
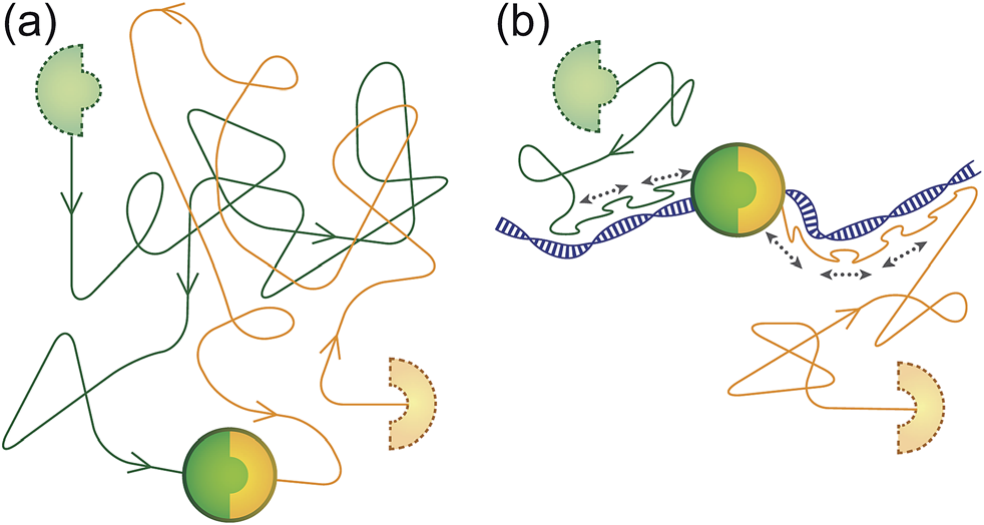
Speeding up association between biomolecules using the ability of a ‘sled’ peptide to one-dimensionally diffuse along DNA. (a) Usually, association between molecules occurs as a result of binding partners finding each other by diffusion in a 3D fashion through solution. (b) Addition of a 1D reaction pathway can drastically speed up the reaction by reducing the dimensionality of search.

**Fig. 2:**
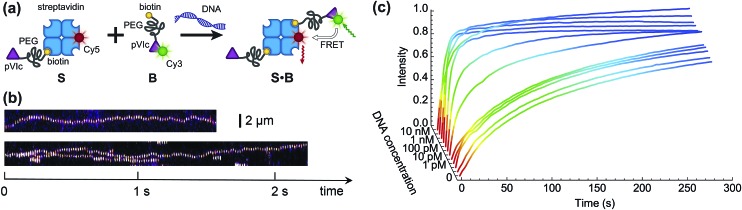
Speeding up bimolecular association by DNA. (a) A schematic of the proof-of-concept biotin–streptavidin system. (b) Single-molecule fluorescence imaging confirms that functionalisation of binding partners with pVIc peptide renders S and B able to 1D slide along DNA. The results are presented as kymographs: top trace shows the sliding of binding partner S, bottom trace shows the sliding of binding partner B, labelled with streptavidin-Cy5 for the ease of detection in view of the signal-to-noise ratio. (c) The formation of complex S·B is monitored in time using various concentrations of 2686 bp long DNA in solution. The time evolution of product concentration can be approximated by exponential growth *C*(*t*) = *C*_max_(1 – e^–*t*/*τ*^), where *τ* is the observed characteristic reaction time.

In this work we demonstrate that the ability of the pVIc peptide to slide along DNA can be used to speed up a much broader class of biomolecular processes than just those occurring *in vivo* and that it can be used to dramatically improve the speed of common laboratory reactions (Fig. 1). First, as a proof of principle, we couple each of the two binding partners in a canonical biotin–streptavidin association to the pVIc ‘molecular sled’ and show that association proceeds more than an order of magnitude faster in the presence of DNA (Fig. 2a). Fluorescence Resonance Energy Transfer (FRET)^12^ was used to monitor the time dependence of the bimolecular association. For simplicity, we refer to the functionalised biotin and streptavidin as binding partners B and S, respectively. Binding partner B is formed by reacting a maleimide-functionalised biotin with the cysteine Cys10 of Cy3-labelled pVIc in a Michael-addition reaction (see ESI, Fig. S1†). The maleimide and biotin units are connected *via* a high-molecular weight polyethylene glycol (PEG) linker resulting in a total molecular weight for binding partner B of 6.7 kDa. This high molecular weight reduces its diffusional mobility and allows us to more easily gain access to the timescale of association. Binding partner S is prepared by forming a complex between a Cy5-labelled tetrameric streptavidin and an unlabelled biotin–pVIc conjugate (see ESI†). The ability of both B and S to 1D diffuse along DNA was confirmed on a single-molecule level using Total Internal Reflection Fluorescence (TIRF) microscopy (Fig. 2b, see ESI† for experimental conditions and notes). We estimated the binding times *τ*_1D_ ≅ 0.3 s and the 1D diffusion coefficient *D*_1D_ ≅ 3 × 10^4^ nm^2^ s^–1^. Using these values, we can calculate that S and B are able to explore a DNA segment of length 
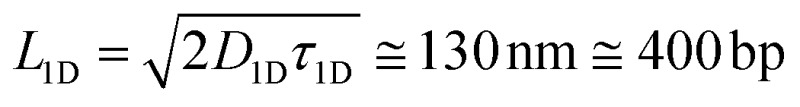
 before dissociating and returning to solution.

Binding partners B and S were combined in aqueous solution at final concentrations of 150 nM and 37.5 nM, respectively, and ensemble FRET between the Cy3 donor and Cy5 acceptor fluorophores was measured (Fig. S2†). Fig. 2c shows the time dependencies of bimolecular association in the presence of 2686 bp long double-stranded DNA (dsDNA) at different concentrations. Addition of the DNA up to 1 pM did not have a significant effect on the reaction rate, whereas DNA concentrations of higher than 10 pM resulted in a clearly discernable reduction of the reaction time. For a DNA concentration of 300 pM, already after 15 s, 99% of the maximum FRET efficiency was achieved.

**Fig. 3:**
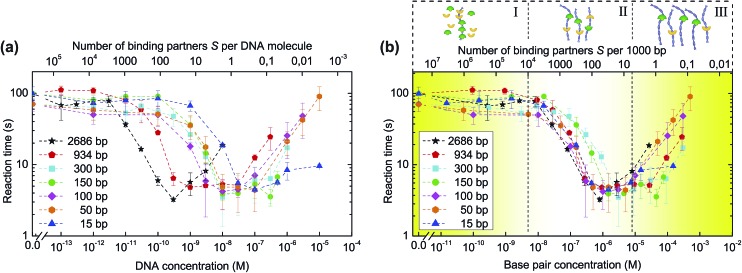
Influence of DNA length and concentration on the reaction time. (a) Biotin–streptavidin association experiments are repeated for different DNA lengths. Reaction times *τ* are presented as a function of DNA concentration. (b) Plotting the dependencies of the reaction times on DNA base pair concentration results in curve clustering together in three regions: (I) no speed-up due to an insufficient number of DNA molecules per binding partner; (II) maximum speed-up with the optimal amount of DNA per binding partner; (III) reduced speed-up caused by the amount of DNA being so high that the probability of binding partners to meet on the same DNA molecule diminishes. Error bars indicate ±SD; *n* ≥ 3.

Fig. 3a shows the reaction times as derived from the FRET traces for different DNA concentrations and lengths. For each length, varying from 2686 to 15 base pairs (Table S1†), the association times decrease by up to 20-fold at higher concentrations of added DNA. Interestingly, the critical concentration for reaction speed up differs for the different DNA lengths: longer DNA fragments are required at lower concentrations than short DNA molecules to achieve the same catalytic effect. This behaviour can be explained by the fact that the critical number of reaction partners associated with DNA is reached at higher DNA concentrations for short fragments and at low DNA concentrations for longer pieces of DNA. Thus, the main parameter that governs the kinetics of reaction is the total base pair concentration, a unit that describes the total length of DNA per unit volume. This notion is validated by plotting the reaction time against DNA base pair concentration (Fig. 3b), showing the curves cluster together in three distinct regions. These regimes can be understood in terms of the density of binding partners trapped on the catalytic DNA molecules. At low base pair concentrations, the amount of DNA available per binding partner is too low to trap a noticeable fraction of the binding partners and influence the overall reaction rate. In the optimal regime, around 0.1 to 10 μM of base pairs, the binding partners have high probability to be trapped by DNA where they can find each other by 1D diffusion. At base pair concentrations higher than 100 μM, the probability for binding partners B and S to bind to the same DNA molecule diminishes, resulting in a deceleration of the association.

In an alternative mechanism to explain the increased association rates, DNA-bound binding partners bound to the same DNA molecule could be brought into proximity of each other by bending and looping of the DNA duplex. In such a mechanism, the binding partners would rely on the conformational flexibility of the DNA and use the duplex as a scaffold to bring binding partners together. In order to exclude this pathway, we conducted a series of experiments with DNA of four different lengths (50, 100, 150 and 300 bp), which were chosen such that the corresponding DNA looping probability differed significantly from one another.^13,14^ Under the low-salt buffer conditions used in this study, DNA molecules of 50 and 100 bp can be regarded as stiff rods whose folding onto itself is excluded (DNA persistence length is estimated to be 250 bp at 2 mM NaCl).^15^ In case looping was the main mechanism for reaction speed-up one would expect a considerably lower reaction acceleration in case of 50 and 100 bp long DNA as compared to 300 bp, which is long enough to form loops. However, in all cases we observed the same 20-fold reaction speed-up (Fig. 3a and b), confirming that association is not mediated by DNA bending onto itself.

Using a similar reasoning, one could argue that the conformational flexibility of the long PEG linkers attached to both binding partners allows those binding partners that are statically but distally bound to the same DNA to associate without the need for sliding. Fig. 3b shows, however, a 10-fold increase in reaction time at a reaction stoichiometry as low as 1 binding partner per 1000 base pairs, clearly an average molecular separation too high to be bridged by the binding partners statically bound to the same DNA. From the considerations above, one can conclude that sliding along DNA, and not just static binding, is responsible for the increase in association rate.

An understanding of the origin of the reaction acceleration effect can be obtained from our recent work in which we formulated a kinetic model for a system with linear sinks (*i.e.* DNA) that can intermittently trap molecules present in a solution and serve as an assembly line for 1D diffusing molecules.^16^ Our model semiquantitatively predicts the experimentally observed speed-up in the presence of DNA molecules of different lengths and concentrations. Moreover, according to our simulations, the relative contribution of the 1D reaction pathway in the optimum speed-up regime can be as high as 90%. In this work, we concluded that although association of the binding partners on DNA without 1D sliding does play a role, the primary contributor to the reaction acceleration is a 1D sliding mechanism. This model also shows that in the case of extremely short DNA molecules (15 bp and 50 bp), the reaction acceleration cannot be explained by 1D sliding alone due to the sizes of the binding partners being comparable to the dimensions of the DNA. Instead, reaction acceleration is introduced by the high diffusional mobility of the short DNA duplexes and their ability to electrostatically capture the cationic peptides.^16^

**Fig. 4:**
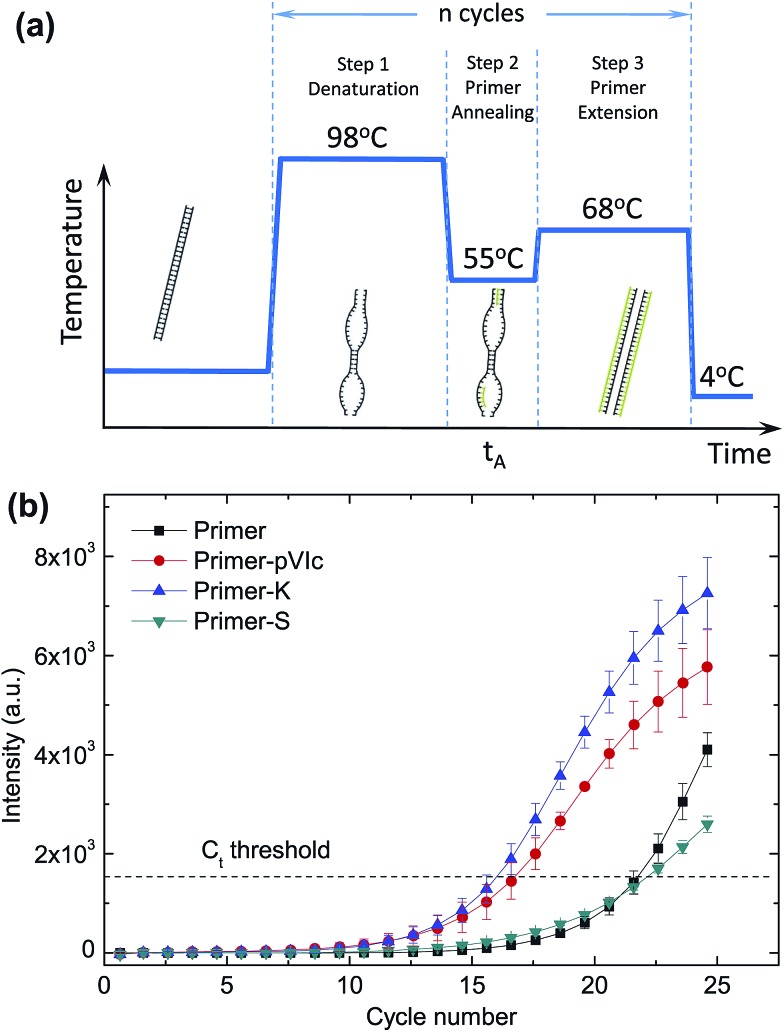
Speed-up of PCR by sled-modified primers. During the annealing step of a thermal cycling PCR protocol (a), primers need to find and hybridise to their complement on a template. This process can be accelerated by attaching the pVIc sled peptide to PCR primers. (b) Amplicon formation for different primer-peptide conjugates is shown for annealing time *t*_A_ = 1 s and primer concentration *C*_primer_ = 0.125 μM. The *C*_t_ threshold values for PCR reactions using unmodified primers, primer-S (negative control), primer-pVIc and primer-K were measured to be 21.7 ± 0.4, 22.1 ± 0.2, 16.7 ± 0.7, and 16 ± 0.5, respectively. Error bars indicate ±SD; *n* = 3.

As a next step, we set out to use our method to speed up a standard polymerase chain reaction (PCR) by reducing the time needed for pVIc-coupled primers to anneal to the template DNA. The exponential amplification of DNA during PCR can be divided into three distinct steps.^17^ The first step is the melting of the double-stranded DNA template (Fig. 4a), followed by primer annealing and elongation with the polymerase. During the annealing step, primers need to find and hybridise to their complementary target sequence on a template. During this annealing step, the DNA will consist of a mixture of denatured and double-stranded regions, providing a large variety of structures for the pVIc–primers to interact with and potentially move along, resulting in a reduction of the time needed for the primer to locate and bind to its target sequence.

We covalently coupled (see ESI†) the pVIc peptide to the 5′ ends of a pair of PCR primers (primer set I, Table S2, Fig. S3 and S4†) designed to amplify a 807 bp stretch from a linear double-stranded 1970 bp-long template and used real-time PCR (qPCR) experiments with SYBR Green I fluorescence to report on the kinetics of amplicon formation^18^ (Fig. 4b and S5†). The correct length of the PCR product was confirmed by agarose gel electrophoresis (Fig. S6†). Similar results were obtained for PCR experiments employing a different pair of primers (Table S2 and Fig. S7†) and a longer 8669 bp-long circular template M13KO7 (Fig. S8†). The kinetics of amplicon formation were quantified in an unbiased manner by employing a PCR threshold cycle analysis (see ESI, Fig. S9†). Remarkably, the PCR reaction containing the pVIc-conjugated primers displayed a significant reduction in the number of cycles needed, suggesting the use of a molecular sled as a viable approach to speed up the overall reaction time of PCR. In our experiments we were able to shorten the PCR reaction time by 15–27%. To ensure that the increase in speed is not caused by a nonspecific electrostatic association between the four positively charged amino acids in the sliding peptide and the negatively charged DNA backbone, we repeated the same PCR experiments using primers conjugated to a scrambled peptide (S-peptide, SFRRCGLRQVK) containing the same residues in a random order, which presumably affects the sliding behaviour of the peptide yet preserving the net charge. Our qPCR data reveals that use of primers conjugated with this scrambled peptide does not result in a decrease of the number of PCR cycles required for amplification (Fig. 4b, ‘S-peptide’).

The performance of the S-peptide-modified primers is very similar to the unmodified primers. Furthermore, we observed a significant reduction in the number of PCR cycles when using a truncated pVIc variant containing only the last six amino acids of pVIc, four of which are positively charged and are sufficient to support sliding along DNA (Fig. 4b, K-peptide, KRRRCF, Fig. S10†). Finally, we studied the behaviour of the primer modifications under different conditions by varying the annealing time *t*_A_ and primer concentration *C*_primer_ (Fig. S8†). In case of the most stringent conditions (short annealing time, low primer concentration) the effect of the sliding peptides was the most pronounced.

To exclude a scenario in which the acceleration effect could originate from the enhanced primer-template binding due to cationic nature of the peptides, we compared the melting temperatures *T*_m_ of the modified and unmodified primers that were used in the PCR experiments. When using short complementary oligonucleotides, and thus excluding sliding contributing to affinity, the measured *T*_m_ values of the peptide-functionalised primers were identical to those of the non-functionalised ones (Fig. S11†). This observation excludes an enhanced stability of binding to DNA in the PCR reactions because of the peptide.

The use of chimeric molecules, where the desired functions of parent moieties are combined within one molecule is a well-established approach in biotechnology. In PCR, for example, attempts have been made to increase the affinity of primers and polymerases to DNA by functionalising primers with DNA-intercalating molecules^19,20^ and expressing the polymerases with an additional cationic peptide motif in the sequence.^21–24^

The enhancement of molecular activity in these cases arises from the increase of the attractive electrostatic and intermolecular forces between the desired molecule and DNA. Another approach that uses the same concept of chimeric molecules is DNA-templated synthesis, where the binding partners are conjugated to single-stranded DNA oligonucleotides and are physically brought into proximity of one another by hybridising them to a DNA template.^25–27^ In our study, however, the mechanism of activity enhancement is different from these approaches: as opposed to increasing the affinity between the binding partners by prolonging the dissociation time, we aimed to speed up association by addition of a different reaction pathway – 1D diffusion along DNA. The reduction of search dimensionality makes the binding partners find each other faster and, thus, results in the overall reaction acceleration.

## Conclusions

Summarising, the 11-a.a. DNA-interacting pVIc peptide acts as a molecular sled in speeding up biochemical reactions by introducing a 1D reaction pathway in addition to bimolecular association *via* 3D diffusion. Our demonstration of the speed up of both a highly generalised reaction and a commonly used laboratory process suggests a wide variety of other potential uses.
