# Supplementary material for: Speeding up biomolecular interactions by molecular sledding
Source: Chem Sci. 2015 Oct 7;7(2):916–20. doi: 10.1039/c5sc03063c (PMC4762599; doi:10.1039/c5sc03063c)
Supplement: Supplementary file 1 [file SC-007-C5SC03063C-s001.pdf]

Electronic Supplementary Material (ESI) for Chemical Science.
This journal is © The Royal Society of Chemistry 2015

Supporting Information

Speeding up biomolecular interactions by molecular sledding

Alexander Turkin,‡[a] Lei Zhang,‡[b] Alessio Marcozzi,[b] Walter F. Mangel,[c] Andreas Herrmann*[b] and Antoine M. van Oijen*[a]

[a]Single-molecule Biophysics, Zernike Institute for Advanced Materials, University of Groningen, Groningen 9747 AG, the Netherlands. E-mail: a.m.van.oijen@rug.nl

[b]Department of Polymer Chemistry, Zernike Institute for Advanced Materials, University of Groningen, Groningen 9747 AG, the Netherlands. E-mail: a.herrmann@rug.nl

[c]Brookhaven National Laboratory, Upton, NY 11973-5000, USA.

‡ These authors contributed equally to this work.

Contents

Materials and methods…………………………………………………………………………...3

Supplemetary figures……………………………………………………………………………….5

Supplemetary tables.………………………………………………………………………16

Additional references……………………………………………………………………………...19

Materials and Methods

Preparation of the binding partners

Stock solution of binding partner B was prepared as follows. N-terminus Cy3 labelled pVIc (custom peptide synthesis by Bio-Synthesis, Inc), 50 μM, was derivatised at Cys10' in 25 mM PBS buffer (pH 7.3) by adding biotin-PEG-maleimide (5 kDa, Nanocs) to 50 μM. The reaction vial was wrapped in aluminium foil to protect from light and the reaction was allowed to proceed for 5 hours at room temperature on a nutator. The reagent was subsequently stored at $+4^0$C and used as is without further purification over the course of one month. Successful conjugation was confirmed by MALDI/TOF mass spectrometry (figure S1).

Spectrofluorometer assay

The experiments were performed in the sliding buffer, containing 60% wt. glycerol, 10 mM HEPES (pH 7.0), 2 mM NaCl, 20 mM Ethanol, 50 μM EDTA. The reactions were run in a 3mL quartz cuvette (FP-1004, Jasco) on a Jasco FP-8300 spectrofluorometer at 20°C, continuously stirred at 800 rpm. The excitation and emission wavelengths were 520 nm and 666 nm correspondingly. The reaction read-out was FRET signal detected as a function of time. The solutions of binding partners *S* and *B* were prepared 30 minutes before the experiment in the following way. Solution 1 (binding partner S), final volume 2 mL, was obtained by incubating Cy5-streptavidin (43-4316, Life Technologies) with a two-fold excess of biotin-PEG23-pVIc (custom peptide synthesis by Bio-Synthesis, Inc) in the sliding buffer to render Cy5-streptavidin molecules functionalised with pVIc. Solution 2 (binding partner B), final volume 1 mL, was prepared from the stock solution and contained biotin-PEG-pVIc-Cy3 in the sliding buffer. Prior to the experiments, DNA of the required length was added at a necessary concentration to both solutions. Solution 1 was transferred into the cuvette and FRET *vs.* time signal acquisition was started. Subsequently, solution 2 was rapidly injected into the cuvette with a syringe. The final reaction mix of a final volume 3 mL contained 37.5 nM Cy5-streptavidin (functionalised by 75 nM biotin-PEG23-pVIc), DNA at a chosen concentration and 150 nM binding partner *B*.

FRET traces analysis

The obtained FRET vs. time traces were initially processed using Spectra Analysis subprogram of the Spectra Manager V.2 software package by JASCO Inc. The data were subject to baseline correction, noise elimination and smoothing (binomial interpolation, 6 iterations). Subsequently, the traces were fit with a single exponential function $C(t) = \mathrm{C}_{max}\left(1 - \mathrm{e}^{-t/\tau}\right)$ and reaction times τ were extracted from the fit.

Single-molecule experiments

The experiments were performed in the sliding buffer (see "spectrofluorometer assay" section) using flow-stretched λ-DNA as a scaffold for sliding. Binding partner *S* was used as is; binding partner *B* was first pre-incubated with Cy5 labelled streptavidin to be able to use a 641 nm (Cy5 excitation) laser instead of a 532 nm (Cy3 excitation) one, which drastically improved the signal-to-noise ratio and allowed us to detect clearly discernible sliding along DNA. The values for binding times $\tau_{1D} \cong 0.3s$ and the 1D diffusion coefficient $D_{1D} \cong 3 \cdot 10^4\, nm^2/s$ (adjusted for high viscosity sliding buffer) of binding partner *S* are based on the studies of P. C. Blainey *et al.*[1]

Primer synthesis

Amino modified primers (provided by biomers.net GmbH, table S2) were functionalised by the peptides in a two-step reaction.[2,3] First, lyophilised amino modified primers (1.5 mM) were reacted with a 20-fold excess of 4-(N-maleimidomethyl)cyclohexane-1-carboxylic acid 3-sulfo-N-hydroxysuccinimide ester (sulfo-SMCC) linker (Sigma Aldrich) in 0.1 M sodium phosphate buffer (pH 7.6). A stock solution of sulfo-SMCC in DMF was used. The reaction was allowed to proceed in a shaker at room temperature overnight. After incubation, the reaction mixtures were centrifuged three times using 3000 Da cut-off spin-columns (Sartorius stedim biotech) to wash out excess Sulfo-SMCC and other small molecules by Milli-Q water. The resulting solution was lyophilised and the conjugate was used for the next coupling step without further treatment. The coupling efficiency was ~50%. Lyophilized primer-SMCC conjugate was dissolved in 0.1 M sodium phosphate buffer (pH 7.6) to obtain a 200 μM solution. Subsequently, dry peptide of choice (pVIc, K or S, provided by CASLO) were added in a 5-fold molar excess. If the peptide did not dissolve completely DMF was added until a clear solution was visible. The reaction mixture was vortexed at room temperature overnight and the product was purified by reversed-phase chromatography (buffer A (pH 7.5): 0.1 M triethylammonium acetate (TEAT) containing 5% Acetonitrile; buffer B (pH 7.5): 0.1 M TEAT containing 65% Acetonitrile; column: RESOURCE RPC 1 mL; gradient: 0-60%, 50CV; wavelength: 260 nm). Finally, the buffer was exchanged to Milli-Q water by centrifugation in a 3000 Da cut-off spin-column. The products were analysed using polyacrylamide gel electrophoresis (PAGE) (figure S3) and MALDI-TOF mass spectrometry (figure S4). For further use the conjugates were lyophilised. In this reaction step, a coupling efficiency of 30 – 40% was achieved.

PCR reactions

Real time PCR (qPCR) experiment was performed using Bio-Rad iQ5 Real-Time PCR System (Bio-Rad Laboratories, Richmond, USA). 20 μL reaction mixtures contained forward and reverse primers (modified or unmodified) at 0.125 μM, DNA template (10 ng for circular 8669-bp M13KO7 DNA template, 5 ng for short linear 1970-bp DNA template), SYBR Green I (1×), Qiagen fast cycling PCR kit (1×) and Q-solution (1×). All reactions (unless indicated otherwise) were performed in triplicate using the following cycling protocol: initial template melting 98°C (5min); 30 cycles of [98°C (1min), 55.6°C (1s), 68°C (30s)]; 68°C (4min), 4°C (hold). The SYBR Green I fluorescence was monitored at the end of each cycle. It should be noted that the primer annealing temperature used was the one calculated for the original unmodified primers. The modified primers may have different optimal annealing temperature but, nevertheless, for all primers the same annealing temperature was used not to introduce a variable in the experiments. To compare the kinetics of amplicon formation for PCR reactions with different primers we employed the threshold cycle (C_t) analysis. The C_t was set in the exponential phase of amplification using the built-in function of the software for Bio-Rad iQ5 Real-Time PCR System. To confirm that no unspecific amplification occurs and that primer annealing step is essential in the PCR protocol we performed several control experiments (figure S5).

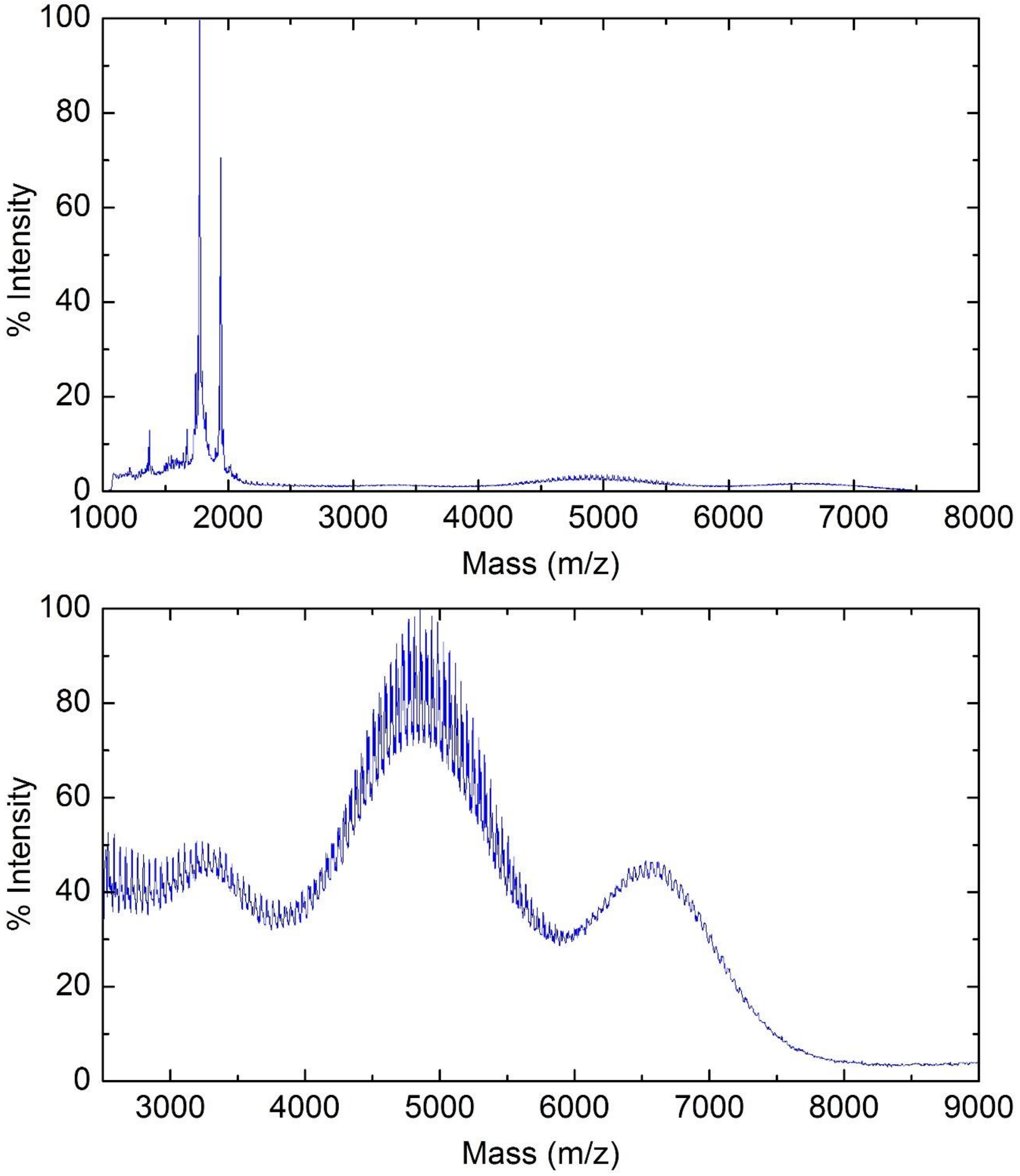

Figure S1. MALDI-TOF mass spectra of binding partner *B*. Binding partner *B* is a conjugate of biotin and Cy3 labelled pVIc. Both units are connected by a poly(ethylene glycol) (PEG) linker, which exhibits a degree of polymerization D_P = 100. Due to the polydisperse nature of the PEG linker a broad mass peak corresponding to binding partner *B* is obtained. The calculated molecular weight (MW) of binding partner *B* is 6700 g/mol, while the the MW determined by MALDI/TOF is 6650 g/mol. After conjugation binding partner *B* was used without further purification because unconjugated Cy3-pVIc would not bind to streptavidin and the unconjugated biotin-PEG while still possessing the ability to bind to streptavidin, would not contribute to the FRET signal.

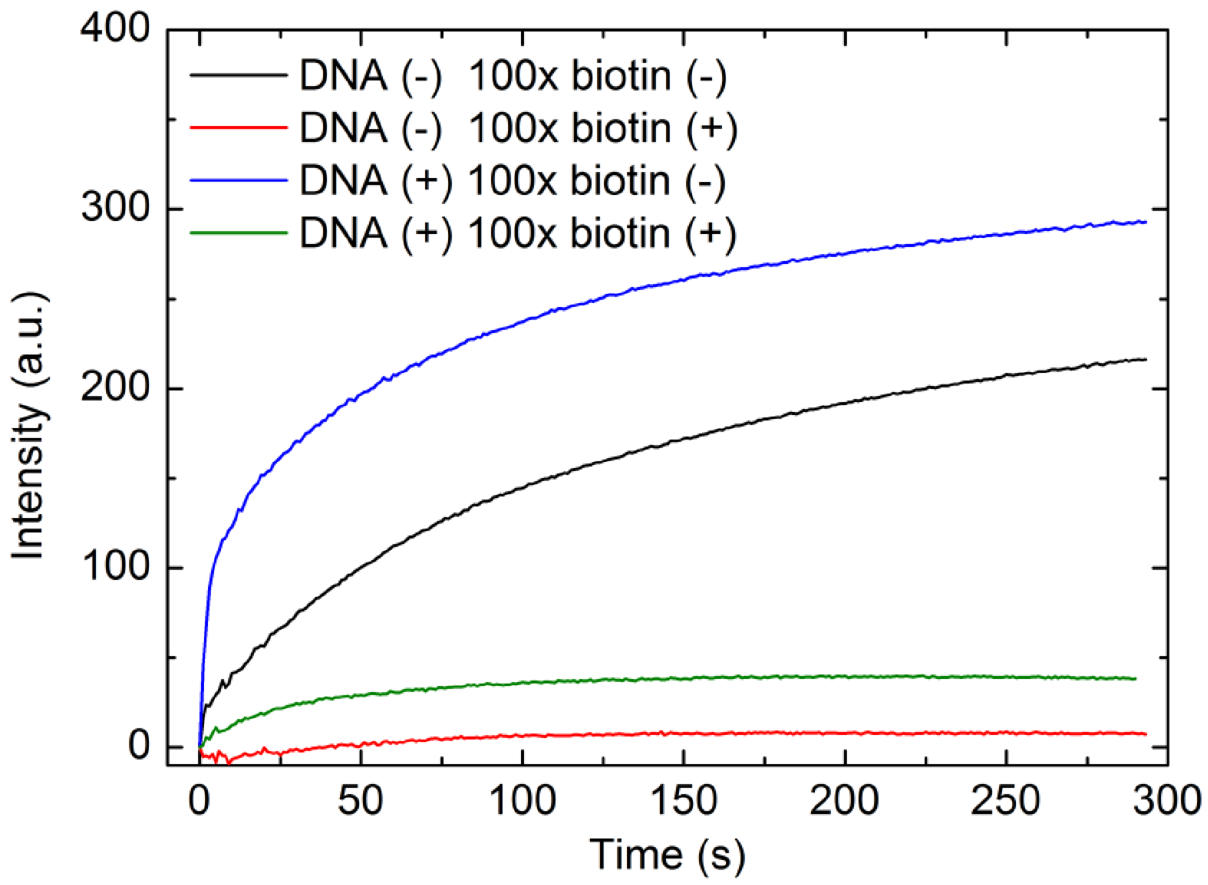

Figure S2. Negative controls for the biotin-streptavidin experiment. To confirm that the rising FRET signal originates from the association between binding partners *S* and *B* we performed the control experiments for which we pre-incubated binding partner *S* with a 100x molar excess of biotin to occupy all binding pockets of streptavidin and prevent binding partner *B* from binding to them. The results for two cases are given: without DNA (black and red curves) and with 10 pM dsDNA of 2686 bp long (blue and green curves). A non-zero FRET signal in case of a 100x biotin excess might be explained by non-specific interactions between binding partners *S* and *B*.

Primer set I

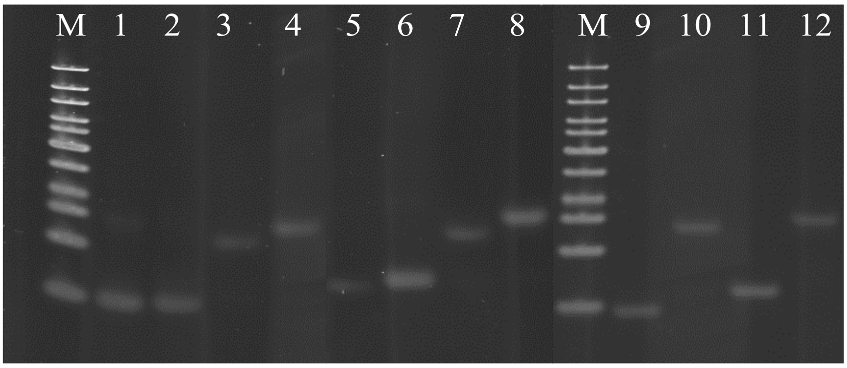

M is DNA ladder for reference (10-300 bp); Lane 1: FP; Lane 2: FP-SMCC linker; Lane 3: FP-K; Lane 4: FP-pVIc; Lane 5: RP; Lane 6: RP-SMCC linker; Lane 7: RP-K; Lane 8: RP-pVIc; Lane 9: FP; Lane 10: FP-S; Lane 11: RP; Lane12: RP-S.

Primer set II

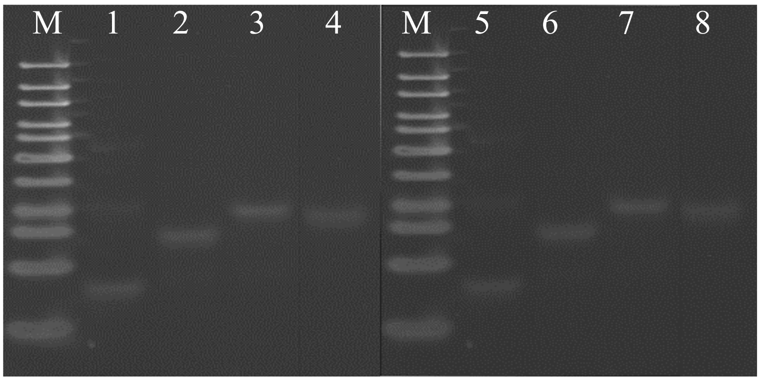

Lane 1: FP; Lane 2: FP-K; Lane 3: FP-pVIc; Lane 4: FP-S; Lane 5: RP; Lane 6: RP-K; Lane 7: RP-pVIc; Lane 8: RP-S.

Figure S3. Analysis of primer-peptide conjugates by polyacrylamide gel electrophoresis (PAGE) after chromatographic purification. The purity of forward (FP) and reverse (RP) primer-peptide conjugates was analysed using a 20% PAGE gel with subsequent SYBR Gold staining.

Primer Set I

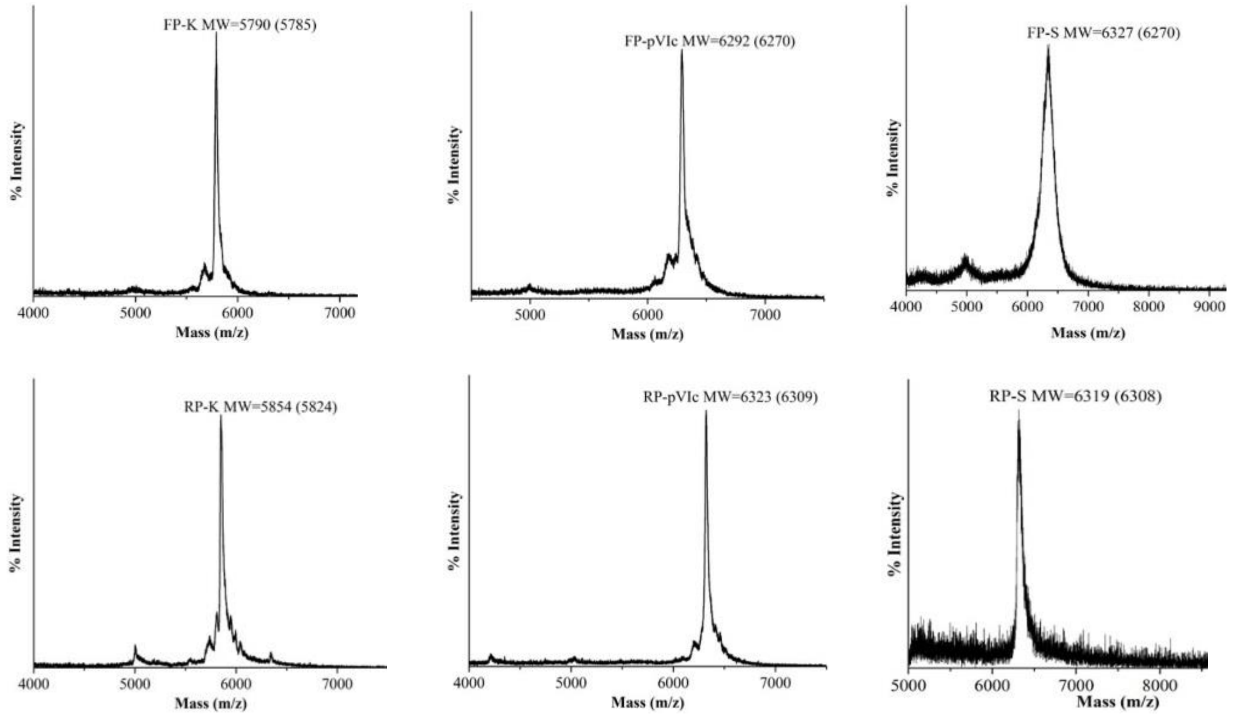

Primer Set II

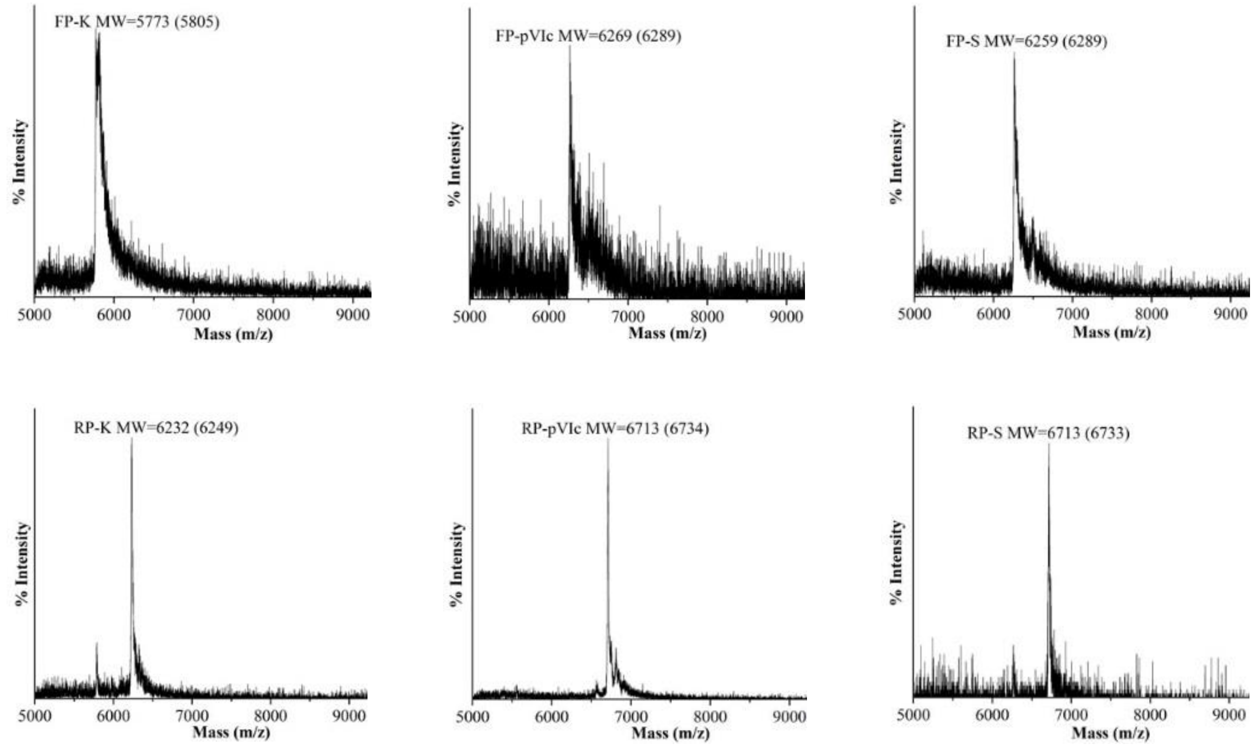

Figure S4. MALDI-TOF mass spectra of primer-peptide conjugates. The molecular weights (MW) obtained from the measurements are shown above the peaks. The predicted MW are given in brackets.

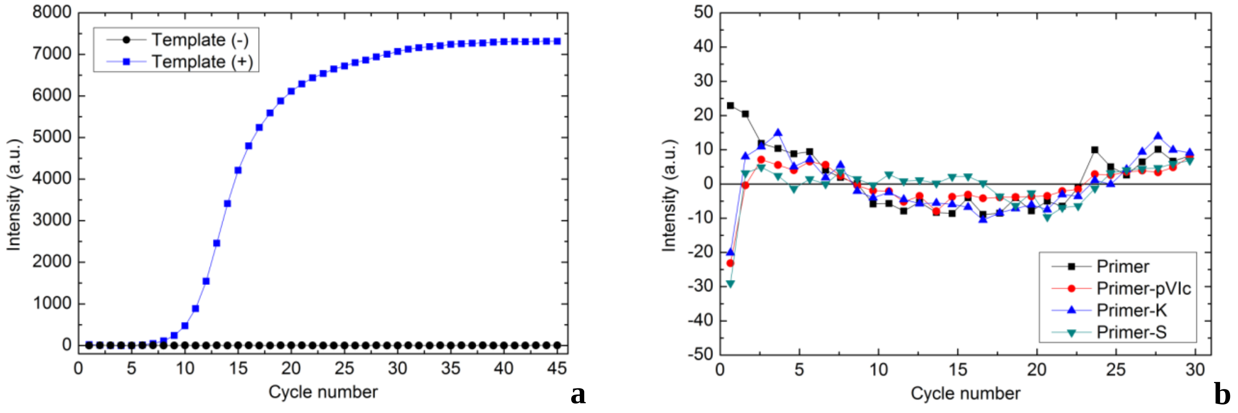

Figure S5. qPCR control experiments. (a) Unmodified primers were used in qPCR with and without DNA template to detect if any unspecific DNA amplification occurred. A 20 µL reaction mixture contained pristine forward and backward primer (primer set I) at a concentration of 0.5 µM, 10 ng DNA template (circular dsDNA of 8669 bp length that originated from bacteriophage M13KO7), Sybr Green I (1×), Qiagen fast cycling PCR kit (1×) and Q-solution (1×). The following qPCR protocol was used: 98°C (5min); 45 cycles of [98°C (1min), 55.6°C (15s), 68°C (30s)]; 68°C (4min), 4°C (hold). When the template was omitted, no amplicon generation was detected indicating that unspecific DNA amplification does not occur. **(b)** To confirm that PCR product formation depends on primer hybridisation on the template and that the annealing of modified and unmodified primers does not occur during the elongation step we performed the qPCR experiment without the annealing step during thermal cycling. The PCR conditions were selected as described above for panel **(a)** except for an altered thermal cycling protocol: 98°C (5min); 30 cycles of [98°C (1min), 68°C (30s)]; 68°C (4min), 4°C (hold). No amplicon formation is detected for any of the primers indicating that the annealing step within the thermal cycling protocol is critical for product formation.

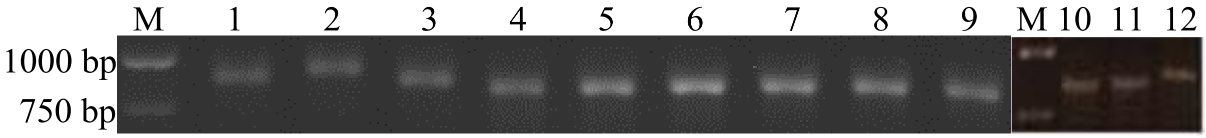

Lane M: DNA ladder; Lane 1-3: amplicon from unmodified primers; Lane 4-6: amplicon from primer-K; Lane 7-9: amplicon from primer-pVIc; Lane 10-12: amplicon from primer-S.

Figure S6. Amplicon formation. The formation of the amplicon of the correct length was confirmed using a 2% agarose gel.

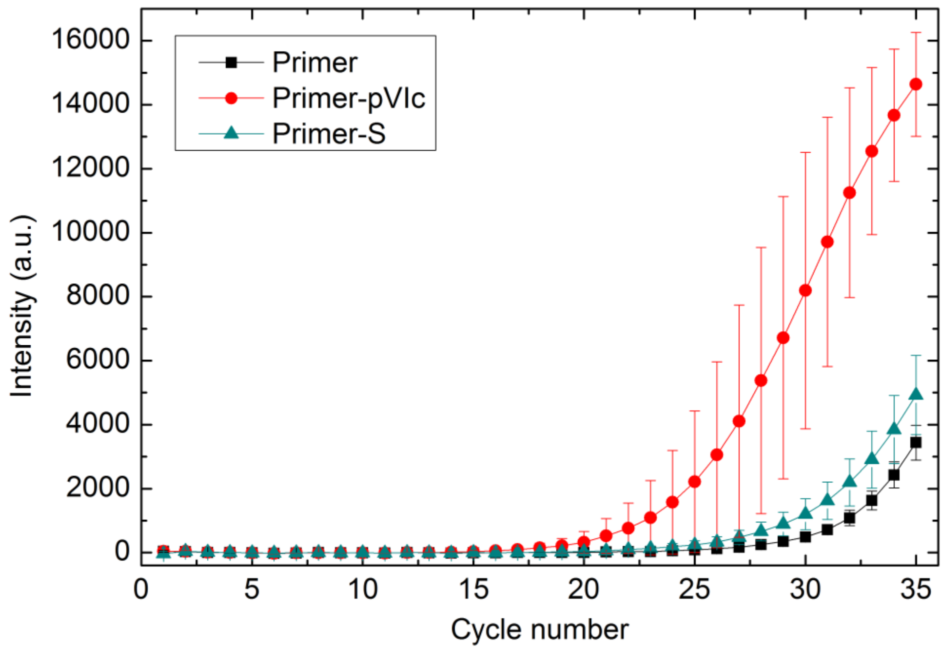

Figure S7. qPCR using primer set II. To prove the general applicability of the sliding peptides to speed up PCR we conducted the qPCR experiments with a different set of primers (Primer set II, table S2). For qPCR conditions and the template see legend to figure S5. Error bars represent standard deviation based on experiments in triplicate.

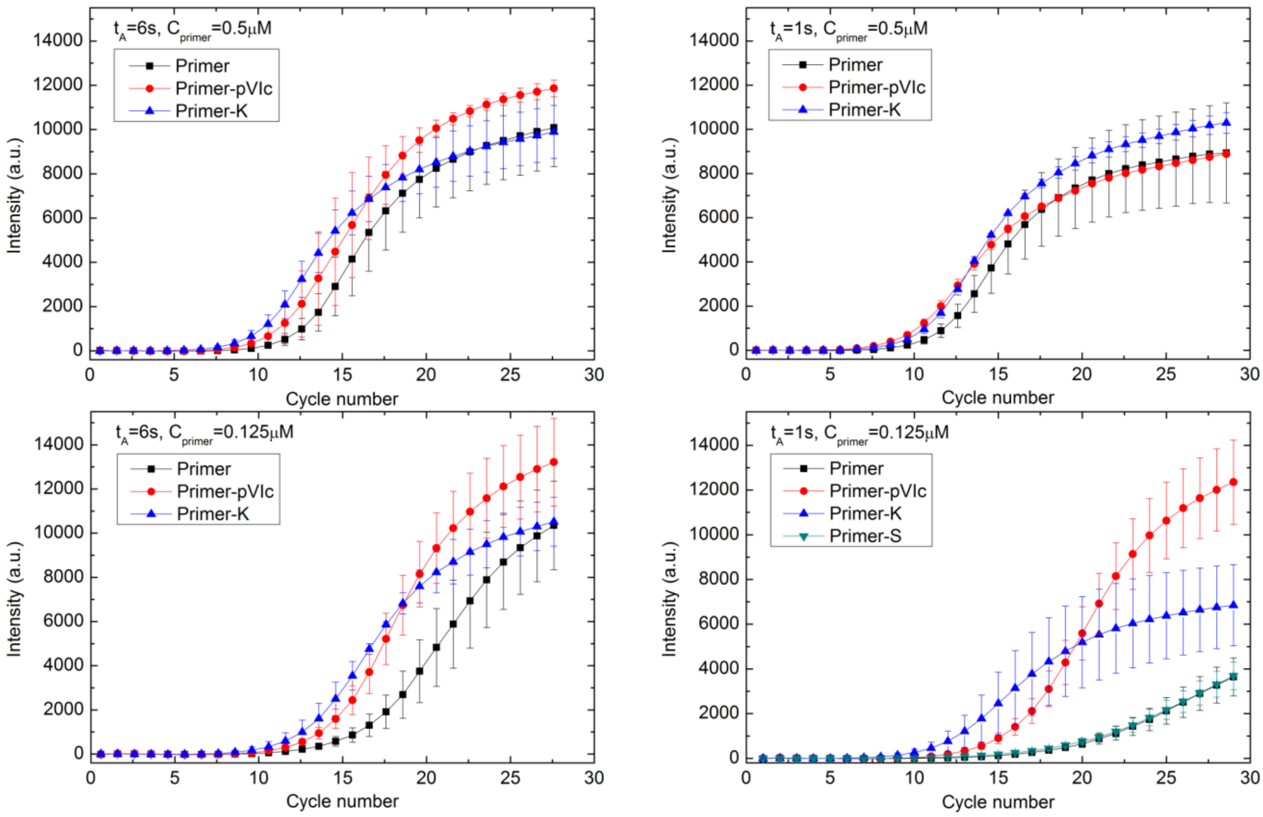

Figure S8. Performance comparison of different primer-peptide modifications under different PCR conditions. The qPCR reactions for primer set I, template 8669 bp dsDNA M13KO7 were performed using different annealing times and primer concentrations. The annealing times in qPCR protocol (for protocol see legend to figure S5) were 1 s and 6 s and two primer concentrations were 0.5 µM and 0.125 µM. Low primer concentrations and low annealing times (the most stringent conditions) gave the highest speed-up of PCR amplification compared to higher annealing times and primer concentration. Error bars represent standard deviation based on experiments in triplicate.

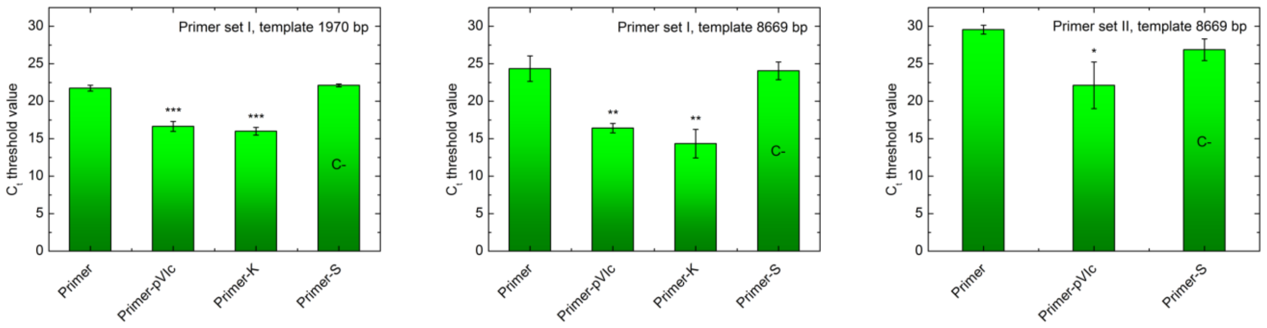

Figure S9. Performance comparison of different primer-peptide conjugates in their ability to speed up the PCR. PCR accelerating effect of the sliding peptides was studied for two different pairs of primers and two different templates. Cycle threshold values C_t were extracted from qPCR traces. Statistical test for significance was unpaired t-test with unequal variance. ***$p<0.001$, **$p<0.01$, *$p<0.05$. Error bars represent standard deviation based on experiments in triplicate.

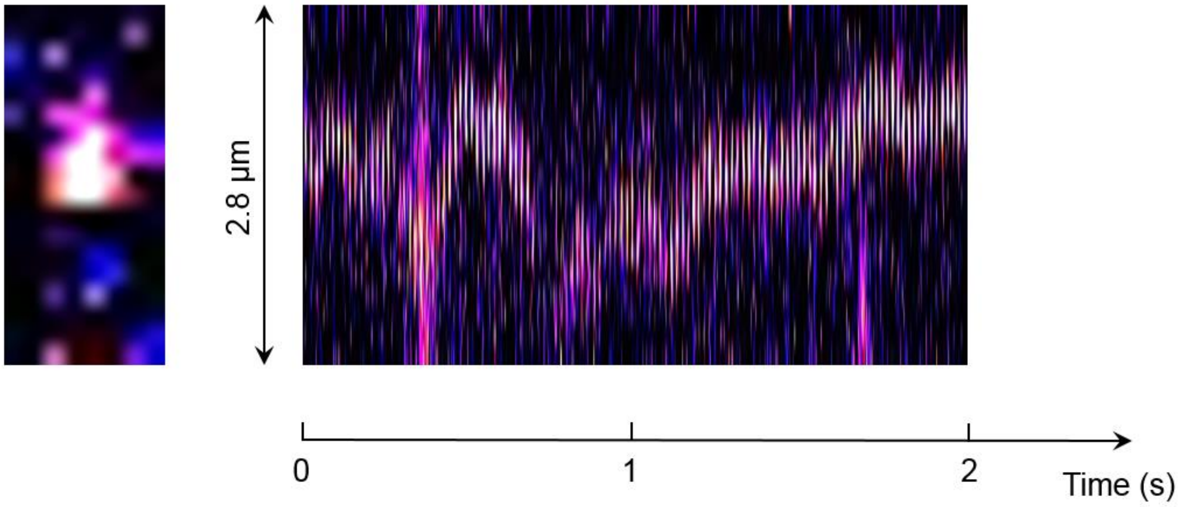

Figure S10. Single-molecule experiments on the sliding of K-peptide. To confirm that peptide K (truncated pVIc) retained the ability to slide, single-molecule experiments were performed on this peptide carrying a fluorophore at the cysteine. The average diffusion coefficient $D_{K-peptide} = 22.12 \cdot 10^6 \, {bp^2}/_{s}$, which corresponds to the values measured for pVIc.

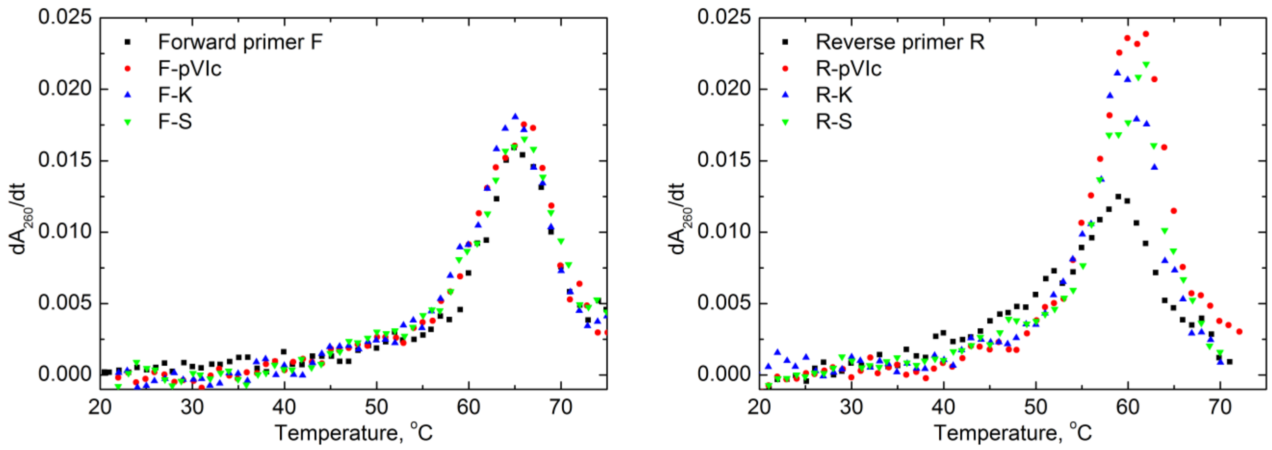

Figure S11. Primer melting temperature measurements. The melting temperatures T_m of the modified and unmodified primers from the primer set II (see table S2) were measured. The absorption at 260 nm was detected while increasing the temperature with a rate of 0.5°C/min starting from 20°C. The measurements were performed using primer-complementary oligo's; the hybridisation buffer was: 10 mM $MgCl_2$, 50 mM NaCl and 10 mM Tris, pH=7.5. The experiments were done in duplicate; the figure shows one set of experiments, the results are depicted as the first derivative of the absorption (260 nm). The obtained T_m values are:

**Primer**	***T_m*, °C**
F	65.5 ± 0.5
F-pVIc	65.5 ± 0.5
F-K	65
F-S	65 ± 1
R	59.5 ± 0.5
R-pVIc	62
R-K	60 ± 1
R-S	61.5 ± 0.5

Table S1. Double stranded DNA sequences used in the proof-of-principle reaction studies.

DNA length	DNA sequence
**15 bp**	5’-GCC TCG CCG CGC CCC-3’<br>(Custom DNA synthesis by Integrated DNA technologies)
**50 bp**	5’-AGA CAG CAT CGG AAC GAG GGT AGC AAC GGC AAC AGA GGC TTT GAG GAC TA-3’<br>(Custom DNA synthesis by Integrated DNA technologies)
**100 bp**	5’-ATG CGC CTG GTC TGT ACA CCG TTC ATC TGT CCT CTT TCA AAG TTG GTC AGT TCG GTT CCC TTA TGA TTG ACC GTC TGC GCC TCG TTC CGG CTA AGT AAC A-3’<br>(Custom DNA synthesis by Integrated DNA technologies)
**150 bp**	5’-TTG AAA AAG CCG TTT CTG TAA TGA AGG AGA AAA CTC ACC GAG GCA GTT CCA TAG GAT GGC AAG ATC CTG GTA TCG GTC TGC GAT TCC GAC TCG TCC AAC ATC AAT ACA ACC TAT TAA TTT CCC CTC GTC AAA AAT AAG GTT ATC AAG TGA-3’<br>(Initially amplified from a kanamycin resistance gene (F primer: 5’-TTG AAA AAG CCG TTT CTG TAA TGA AGG AGA AAA CTC-3’, R-primer: 5’-TCA CTT GAT AAC CTT ATT TTT GAC GAG GGG AAA TTA-3’) Subsequently amplified using 150mer itself as a template. Purified by ethanol precipitation)
**300 bp**	5’-AGT GCG ATT AAG CCA GAC ATG AAG ATC AAA CTC CGT ATG GAA GGC AAC GTA AAC GGG CAC CAC TTT GTG ATC GAC GGA GAT GGT ACA GGC AAG CCT TTT GAG GGA AAA CAG AGT ATG GAT CTT GAA GTC AAA GAG GGC GGA CCT CTG CCT TTT GCC TTT GAT ATC CTG ACC ACT GCA TTC CAT TAC GGC AAC AGG GTA TTC GCC AAA TAT CCA GAC AAC ATA CAA GAC TAT TTT AAG CAG TCG TTT CCT AAG GGG TAT TCG TGG GAA CGA AGC TTG ACT TTC GAA GAC GGG GGC ATT TGC-3’<br>(Initially amplified from pNZ-mEos3.2 plasmid (F primer: 5’- AGT GCG ATT AAG CCA GAC ATG AAG ATC AAA CTC CGT-3’, R primer: 5’- GCA AAT GCC CCC GTC TTC GAA AGT CAA GCT TCG T-3’) Subsequently amplified using 300mer itself as a template. Purified by ethanol precipitation)

**934 bp**	5'-CGC GAG CAT AAT AAA CGG CTC TGA TTA AAT TCT GAA GTT TGT TAG ATA CAA TGA TTT CGT TCG AAG GAA CTA CAA AAT AAA TTA TAA GGA GGC ACT CAC CAT GGG AAG TGC GAT TAA GCC AGA CAT GAA GAT CAA ACT CCG TAT GGA AGG CAA CGT AAA CGG GCA CCA CTT TGT GAT CGA CGG AGA TGG TAC AGG CAA GCC TTT GAG GGA AAA CAG AGT ATG GAT CTT GAA GTC AAA GAG GGC GGA CCT CTG CCT TTT GCC TTT GAT ATC CTG ACC ACT GCA TTC CAT TAC GGC AAC AGG GTA TTC GCC AAA TAT CCA GAC AAC ATA CAA GAC TAT TTT AAG CAG TCG TTT CCT AAG GGG TAT TCG TGG GAA CGA AGC TTG ACT TTC GAA GAC GGG GGC ATT TGC AAC GCC AGA AAC GAC ATA ACA ATG GAA GGG GAC ACT TTC TAT AAT AAA GTT CGA TTT TAT GGT ACC AAC TTT CCC GCC AAT GGT CCA GTT ATG CAG AAG AAG ACG CTG AAA TGG GAG CCC TCC ACT GAG AAA ATG TAT GTG CGT GAT GGA GTG CTG ACG GGT GAT ATT GAG ATG GCT TTG TTG CTT GAA GGA AAT GCC CAT TAC CGA TGT GAC TTC AGA ACT ACT TAC AAA GCT AAG GAG AAG GGT GTC AAG TTA CCA GGC GCC CAC TTT GTG GAC CAC TGC ATT GAG ATT TTA AGC CAT GAC AAA GAT TAC AAC AAG GTT AAG CTG TAT GAG CAT GCT GTT GCT CAT TCT GGA TTG CCT GAC AAT GCC AGA CGA TAA CTG CAG GCA TGC GGT ACC ACT AGT TCT AGA GAG CTC AAG CTT TCT TTG AAC CAA AAT TAG AAA ACC AAG GCT TGA AAC GTT CAA TTG AAA TGG CAA TTA AAC AAA TTA CAG CAC GTG TTG CTT TGA TTG ATA GCC AAA AAG CAG CAG TTG-3'<br>(Initially amplified from pNZ-mEos3.2 plasmid (F primer: 5'-CGC GAG CAT AAT AAA CGG CTC TG-3', R primer: 5'-CAA CTG CTG CTT TTT GGC TAT C-3') Subsequently amplified using 934mer itself as a template. Purified by ethanol precipitation)
**2686 bp**	pUC19 vector<br>(N3041L, New England Biolabs)

Table S2. Primer sets used in PCR experiments.

Primer set I	Forward:<br>Reverse:	5’-(NH_2)-CTC ATC GAG CAT CAA-3’<br>5’-(NH_2)-ATG AGC CAT ATT CAA-3’	807 bp amplicon
Primer set II	Forward:<br>Reverse:	5’-(NH_2)-GCG TTT CCT CGG TTT-3’<br>5’-(NH_2)-GTA ATT TAG GCA GAG G-3’	831 bp amplicon

References:

1. P. C. Blainey, V. Graziano, A. J. Péres-Berná, W. J. McGrath, S. Jane Flint, C. San Martín, X. Sunney Xie and W. F. Mangel, *The J. of Biol. Chem.*, 2013, **288**, 2092-2102.
2. C.H. Tung, M.J. Rudolph, S. Stein, *Bioconj. Chem.*, 1991, **2**, 464-465.
3. A.W. Fraley *et al.*, *J. Am. Chem. Soc.*, 2006, **128**, 10763-10771.
